# Identifying Alzheimer’s Disease Progression Subphenotypes via a Graph-based Framework using Electronic Health Records

**DOI:** 10.21203/rs.3.rs-6257332/v1

**Published:** 2025-04-07

**Authors:** Yu Huang, Jie Xu, Zhengkang Fan, Yu Hu, Xing He, Aokun Chen, Yuxi Liu, Rui Yin, Jingchuan Guo, Steven T. DeKosky, Michael Jaffee, Manqi Zhou, Chang Su, Fei Wang, Yi Guo, Jiang Bian

**Affiliations:** Indiana University; University of Florida; University of Florida; University of Florida; Indiana University; University of Florida; Indiana University; University of Florida; University of Florida; University of Florida; University of Florida; Cornell University; Weill Cornell Medicine; Weill Cornell Medicine; University of Florida; Indiana University

**Keywords:** Alzheimer’s Disease, Disease Progression Subphenotyping, Real-world Data, Graph Neural Network, Electronic Health Records

## Abstract

**Purpose:**

Understanding the heterogeneity of neurodegeneration in Alzheimer’s disease (AD) development, as well as identifying AD progression pathways, is vital for enhancing diagnosis, treatment, prognosis, and prevention strategies. To identify disease progression subphenotypes in patients with mild cognitive impairment (MCI) and AD using electronic health records (EHRs).

**Methods:**

We identified patients with mild cognitive impairment (MCI) and AD from the electronic health records from the OneFlorida+ Clinical Research Consortium. We proposed an outcome-oriented graph neural network-based model to identify progression pathways from MCI to AD.

**Results:**

Of the included 2,525 patients, 61.66% were female, and the mean age was 76. In this cohort, 64.83% were Non-Hispanic White (NHW), 16.48% were Non-Hispanic Black (NHB), and 2.53% were of other races. Additionally, there were 274 Hispanic patients, accounting for 10.85% of the total patient population. The average duration from the first MCI diagnosis to the transition to AD was 891 days. We identified four progression subphenotypes, each with distinct characteristics. The average progression times from MCI to AD varied among these subphenotypes, ranging from 805 to 1,236 days.

**Conclusion:**

The findings suggest that AD does not follow uniform transitions of disease states but rather exhibits heterogeneous progression pathways. Our proposed framework holds the potential to identify AD progression subphenotypes, providing valuable and explainable insights for the development of the disease.

## Introduction

Alzheimer’s disease (AD) is the most common form of dementia, characterized by a gradual and irreversible decline in cognitive function. This heterogeneous aging-related neurodegenerative disorder affects a large number (approximately 1 in 9 people age 65) of older adults globally.^1^ As of 2023, an estimated 6.7 million Americans are living with AD, and this number is projected to increase to 13.85 million by 2060.^1,2^ The growing population of individuals affected by AD will undoubtedly impose a substantial burden on patients, their families, the healthcare system, and society at large, presenting a critical problem that requires careful consideration and attention.

The AD continuum, which describes the progression of AD, starts with brain changes that often go unnoticed and advances in the majority of cases to memory difficulties, ultimately leading to significant cognitive and functional impairments. This progression is commonly hypothesized to encompass three broad phases: preclinical AD, clinically significant mild cognitive impairment (MCI) due to AD, and Alzheimer’s dementia.^3–5^ The duration of each phase of AD varies among individuals, influenced by factors such as demographics, genetics, environmental factors, and lifestyle, leading to heterogeneous clinical outcomes.^6^ The rate of cognitive decline varies widely among AD patients; some experience rapid deterioration while others show slower rates of decline over time.^7^ Therefore, understanding the variety of progression patterns within the AD continuum and the ability to identify early diagnostic indicators are of significant clinical importance.

Recent studies have focused on incorporating diverse data sources to study the development of AD, including clinical variables,^8^ neuroimaging,^9,10^ neuropsychological,^11,12^ and neuropathological data,^13^ aiming to identify clinical predictors and biomarkers^14–16^ that can be used to track disease changes.^17^ Disease subphenotyping that segments patients diagnosed with the same disease into multiple subentities (subphenotypes), with unique clinical manifestations, phenotypic progression trajectories, and/or clinical outcomes, has been attracting increasing attention in biomedicine to study complex diseases like AD.^18^ Existing studies have often focused on a restricted range of features, such as neuroimaging,^19^ neuropsychological data,^12^ and neuropathological data,^13^ to identify subphenotypes of AD patients. However, some of these data sources (e.g., neuroimaging biomarkers), require specialized and costly equipment that are not widely available. This limitation restricts their use in routine clinical care in less well-equipped settings and constrains the sample size available for subphenotyping analyses.

The proliferation of accessible real-world data (RWD), especially electronic health records (EHRs) and administrative claims data, coupled with advancements in artificial intelligence (AI) and machine learning (ML) techniques, has opened new avenues for investigating the heterogeneity of AD progression in a data-driven manner.^20^ Longitudinal EHRs collected during routine care provide fine-grained encounter information, enabling tracking changes in the health status of AD patients. These records are valuable for identifying AD subphenotypes, yet few studies have focused on utilizing them for this purpose.^21–25^ Most existing approaches do not consider relevant clinical outcomes, such as disease severity, leading to limited utility for clinicians and patients. Additionally, these methods have struggled to capture progression characteristics in the AD continuum, and they have been limited in modeling the similarity of progression trends and patterns among patients. Therefore, it is necessary to model the underlying relationships among patient encounters with the health systems and introduce outcome-oriented learning manners^26^ to identify subgroups (i.e., subphenotypes) with homogeneous progression and clinical characteristics that are strongly associated with future clinical outcomes.

We developed a computational approach, i.e., a novel graph neural network (GNN)-based framework. This framework leverages a directed patient graph (DPG) to model patient longitudinal EHRs, connecting visits with similar clinical characteristics and enabling GNNs to effectively learn patient progression patterns. We incorporated an outcome-driven strategy to guide the GNN, ensuring that the extracted embeddings are clinically relevant to the progression from MCI to AD. Additionally, this framework utilizes time series clustering to analyze sequential embeddings learned from GNNs, identifying MCI to AD progression subphenotypes (i.e., pathways). Through a series of experiments conducted on a large-scale EHR cohort from the OneFlorida + Clinical Research Consortium, we demonstrated the presence of specific progression pathways leading to AD. This study fills important gaps in understanding the heterogeneity of AD progression, which can potentially enhance earlier diagnosis and intervention for AD.

## Methods

### Data source and study population

**Figure 1:**
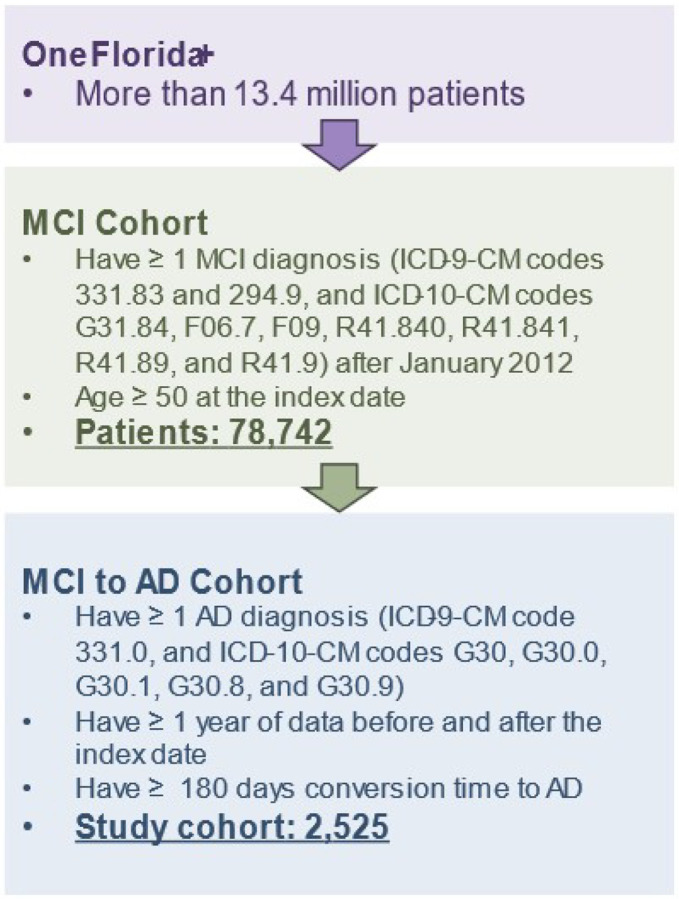
Overview of the study cohort extracted from the OneFlorida+ network.

In this study, we used RWD from OneFlorida+, a clinical research network contributing to the national Patient-Centered Clinical Research Network (PCORnet), which collaborates with a group of 14 health organizations. OneFlorida + contains robust, longitudinal, patient-level EHRs of 16.8 million patients from Florida, 2.1 million from Georgia, and 1.1 million from Alabama, and is linked at the patient level with various other data sources, including selected Medicaid and Medicare claims, vital statistics, and tumor registries. OneFlorida + is a HIPAA-limited data set (i.e., dates and 9-digit zip codes are available) following the PCORnet Common Data Model (CDM) and contains detailed patient and clinical variables, including demographics, vital signs, conditions, diagnoses, procedures, prescribing, dispensing, and lab results, among others, covering data from 2012 to present. Figure 1 shows an overview of the study cohort construction process. The study cohort includes patients who: (1) had a diagnosis of MCI (International Classification of Diseases, Ninth Revision, Clinical Modification [ICD-9-CM] codes 331.83 and 294.9, and International Classification of Diseases, Tenth Revision, Clinical Modification [ICD-10-CM] codes G31.84, F06.7, F09, R41.840, R41.841, R41.89, and R41.9), and (2) were 50 years or older at the time of their first MCI diagnosis. We further identified patients with AD within the MCI cohort using ICD-9-CM code 331.0 and ICD-10-CM codes G30, G30.0, G30.1, G30.8, and G30.9. Those who had AD diagnosis codes before their MCI diagnosis were excluded from the study. To ensure a sufficient duration of data for training the proposed framework, we imposed two additional inclusion criteria: (1) patients were required to have at least one year of data before and after the index date, and (2) patients were required to have a conversion time to the AD of more than six months.

### Disease progression subphenotyping framework

**Figure 2:**
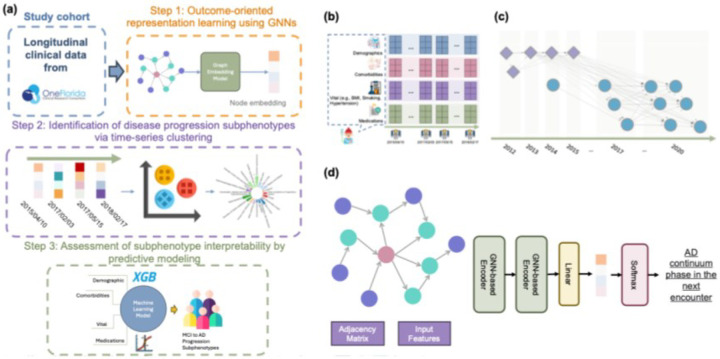
(a) Study pipeline; (b) exemplar EHR sequence of a patient; (c) an example of the proposed Disease Progression Graph (DPG). Each node represents an encounter; the shape and color of a node differentiates the patient, and the dotted lines represent edges that link nodes of different patients; (d) GNN-based framework for learning embedding for a single data point.

Figure 2-a illustrates the proposed framework, which consists of three components: (1) Outcome-oriented representation learning using GNNs, (2) Identification of disease progression subphenotypes via time-series clustering, and (3) Assessment of subphenotype interpretability by predictive modeling.

### Step 1: Outcome-oriented representation learning using GNNs

The longitudinal EHRs of the n-th patient can be represented as $\left\{x_{t}^{n}\right\}$, where t∈{1,2,…,T}, and $x_{t}^{n}$ contains multi-source information (e.g., demographics, diagnoses, and treatments) documented at each visit (encounter). For each encounter, we first discretized age using uniform-sized bins and utilized one-hot encoding to encode variables such as age, gender, and race-ethnicity.^27^ We then mapped the diagnosis codes to Phecodes,^28^ which are designed to support phenome-wide association studies (PheWAS) in EHRs, and transformed the drug codes (e.g., National Drug Codes [NDC] and RxNorm) to the third level of the Anatomical Therapeutic Chemical (ATC) Classification System. We discretized the Body Mass Index (BMI) into four categories: underweight (≤ 18.5), normal weight (18.5–24.9), overweight (25–29.9), and obesity (≥ 30),^29^ the blood pressure readings to five classes (normal, elevated, hypertension stage 1, hypertension stage 2, and hypertensive crisis),^30^ and smoking status to current, former, non-smoker, and others. Finally, we formed a binary vector by concatenating the encoded age, gender, and race from the encounters with diagnoses and medications in a three-month period up to time point t for each patient, resulting in an enhanced encounter representation ${\hat{x}}_{t}^{n}$.

Then, we modeled the processed longitudinal EHRs into a novel disease progression graph (DPG). This graph structure effectively captures individual patient progression patterns and preserves the inter-patient progression correlations, as depicted in Fig. 2-b. Formally, we defined the DPG as a directed graph, DPG=(𝒱,ℰ,𝒜), where each node v∈𝒱 represents an enhanced encounter representation ${\hat{x}}_{t}^{n}$, incorporating patient characteristics (e.g., demographics, diagnoses, vital information, and treatments). To construct the DPG, we began by identifying the top

k most similar neighbors for each node, utilizing a similarity function based on the Jaccard index:

$$S\left(F_{v},F_{u}\right)=\frac{\left|F_{v}\cap F_{u}\right|}{\left|F_{v}\cup F_{u}\right|}$$

Subsequently, we inserted a directed edge e∈ℰ between each pair of similar nodes, with the direction reflecting the chronological order of the encounters. In addition, we checked and inserted edges between nodes (i.e., enhanced encounter representation) from the same patient to ensure that the progression of the disease can be linked through a path. The adjacency matrix 𝒜 captures the weights of the edges (i.e., the elapsed time between two encounters). Figure 2-c shows an example of the proposed DPG.

To generate outcome-oriented embeddings for each node, we developed an encoder based on GNNs as shown in Fig. 2-d. This encoder utilizes the graph structures to propagate and aggregate node features in an iterative manner. The encoder contains two GNN layers and a fully connected layer to transform the learned embeddings to a specific label. The GNN layers^31^ can be described as:

$$h_{v}=\varphi \left(F_{v},\underset{u\in N_{v}}{⨁}\psi \left(F_{v},F_{u},a_{v,u}\right)\right)$$

where $h_{v}$ refers to the learned embeddings for a node v,φ and ψ are learnable functions, ⨁ is nonparametric operation (e.g., aggregation and concatenation), $N_{v}$ means the neighbors of v, and $a_{v,u}$ is the weight of edge between nodes v and u. We utilized MagNet^32^, graph convolutional network (GCN) designed for directed graphs, as the core of the GNN layer. MagNet extends traditional GCNs by incorporating directional information via a complex Hermitian matrix. With its superior ability to encode structural information from directed graphs and outperforming traditional GCNs on various benchmarks, Magnet is an excellent candidate for modeling the intricate information of disease progression contained within DPGs. Additionally, we implemented other three variants of GNNs, including GCN,^33^ graph attention network (GAT),^34^ and graph sample and aggregate network (GraphSAGE)^35^ to compare their performance in embedding learning.

The embeddings for each node are generated by GNN layers, capturing the underlying disease progression information relevant to patient encounters. These outcome-oriented embeddings are then fed into the fully connected predictor, which forecasts the outcomes of the next encounter along the AD continuum: preclinical, MCI, and AD. The fully connected layer can be formulated as:

$$y=\theta \left(Wh_{u}+b\right)$$

where θ is the activation function and W and b are the learnable parameters. During the training stage, we employed gradient descent to optimize the GNNs by minimizing the difference between the actual and predicted labels.

### Step 2: Identification of disease progression subphenotypes via time-series clustering

After obtaining the learned embeddings of each node (i.e., enhanced encounter representation), we combined these embeddings into a sequence (forming a multivariate time series). Each patient had an embedding sequence arranged in chronological order based on the original longitudinal EHRs, described as $H_{\text{T}}^{n}=\left\{h_{1}^{n},\ldots ,h_{t}^{n}\right\}$, where n and T are patient and time index, respectively. We then applied the time-series K-means clustering method with dynamic time warping (DTW)^36^ to identify similar latent characteristics among embedding sequences and determine progression subphenotypes. Time series k-means clusters similar time series data by grouping them based on their similarity. This iterative algorithm repeatedly reassigns time series to clusters and updates the centroids to minimize the within-cluster errors, as defined:

$$J=\sum_{i=1}^{K}\sum_{j=1}^{C_{i}}DTW\left(H_{\left(j\right)}^{\left(i\right)},\mu_{i}\right)$$

where K is the number of clusters, $C_{i}$ refers to the number of patients in cluster $i,H_{\left(j\right)}^{\left(i\right)}$ is the embedding sequence of the j-th patient in cluster i, and $\mu_{i}$ denotes the centroid of cluster i. The function DTW is a distance metric used to measure similarity between two embedding sequences of different length. To do so, the main idea is to find an optimal warping path with the minimum distance between the sequences $S=\left\{S_{1},\ldots ,S_{N}\right\}$ and $S\prime =\left\{S\prime_{1},\ldots ,S\prime_{M}\right\}$, the idea is to find. A warping path, also called an (N,M) -warping path is defined as a sequence $P=\left\{p_{1},\ldots p_{L}\right\}$, with $p_{l}=\left(S_{l},S\prime_{l}\right)\in [1:N]\times [1:M]$ for l∈[1:L]. Formally, the optimization problem can be expressed as:

$$D_{P}≔\sum_{i=1}^{L\in \;P}d\left(S_{i},S\prime_{i}\right)$$

$$DTW\left(S,S^{\prime }\right)≔D_{P*}(S,S\prime )=\underset{P\in D\left(S,S^{\prime }\right)}{\text{min}}\left\{D_{P}\left(S,S^{\prime }\right)\right\}$$

where d() is the distance function (e.g., Euclidian distance) between two data points in different sequences. D(S,S′) is the set of all possible paths, and P* is the optimial path.

To determine the optimal K, we adopted a combination of quantitative and qualitative analyses. Specifically, we applied the time series K-means algorithm with varying cluster numbers (K = 2 to 10) to generate clustering outcomes under different K settings. Then, we used silhouette score (SS)^37^ and Davies-Bouldin Index (DBI)^38^, with DTW as the similarity metrics, to measure the quality of clusters. Clustering results were considered as acceptable if SS is above 0.25 and DBI was below 1.^39^ After we obtain the candidate results (e.g., K from 2 to 4 in Magnet showing fair and acceptable quantative performance), we examined the characteristics of each cluster under different settings and manually assessed cluster quality. This assessment considered factors include (1) variations in the transition time from MCI to AD, (2) differences in survival time post-AD diagnosis, and (3) comorbidities, medications, and demographics. Two reviewers (Y.H. and J.X.) evaluated the cluster quality, and a consensus discussion, led by a third reviewer (J.B.), was conducted to consolidate opinions and determine the optimal K.^40^ Subsequently, we used the time series K-means algorithm again, now with the optimal K, to classify the embedding sequence for each patient into distinct clusters, where each cluster describes a unique disease progression subphenotype. We chose time series K-means due to its simplicity as an unsupervised algorithm, known for its rapid convergence, even on large datasets.

### Step 3: Assessment of subphenotype interpretability by predictive modeling

To ensure that the subphenotypes were clinically useful, we built prediction models to assess their predictability. We first generated the subphenotypes based on the proposed GNN framework for each patient. Then, we set the prediction index date as the patient’s MCI diagnosis date and used information (e.g., demographics, comorbidities, and medications) from the first visit up to the index date to predict which subphenotype the patient belonged to. We implemented various commonly used ML models for this purpose, including linear models (e.g., logistic regression,^41^ lasso regression,^42^ ridge regression,^43^ and ElasticNet^44^) and XGBoost.^45–50^ We also incorporated two imblanced data preprocessing methods, random over sampling and random under sampling. Finally, we utilized SHapley Additive exPlanations (SHAP)^51^ – a widely used XAI technique – to identify important features contributing to the models’ ability to classify the subphenotype an encounter belonged to.

### Modeling procedures and benchmarks

We followed ML best practices, stratified the data by patients, and split it into training, validation, and testing sets according to a 70%:10%:20% ratio. We selected the Area under the Receiver Operating Characteristic Curve (AUROC) as our primary metric and included sensitivity, specificity, and precision as additional metrics for the GNNs and the subphenotype prediction model. Furthermore, we conducted a five-fold cross-validation Bayesian search on the training set to optimize the parameters of the subphenotype prediction models.

## Results

### Descriptive statistics of the study cohort

Our final analysis included 2,525 eligible MCI and AD patients in the cohort. **eTable 1** highlights the characteristics of the study cohort. The mean age of the patients was 76 (std = 8.88), with 61.66% being women. In the cohort, 64.83% were Non-Hispanic White (NHW), 16.48% were Non-Hispanic Black (NHB), and 2.53% were of other races. Additionally, there were 274 Hispanic patients, accounting for 10.85% of the total patient population. The average duration from their first MCI diagnosis to transition to AD was 891 days.

### GNN performance for learning disease progression representation

**eTables 2 to 5** present the performance analysis of four GNNs under different settings, including the number of neighbors (n = 25,50,100,200) in DPG, choice of loss functions (e.g., cross-entropy or Focal loss^52^) and the embedding size (e.g., 32 or 64) for learning outcome-oriented disease progression representations. The GCN model shows the lowest performance, with all metrics around or below 0.9. In contrast, MagNet, GAT, and GraphSAGE demonstrated exemplary performance, with consistently high precision, recall, and specificity rates, all exceeding 0.95. Notably, increasing the dimensionality of the embeddings correlated with a slight improvement in model prediction performance. Among the two loss functions, Focal loss achieved better performance metrics.

### Identifying AD progression subphenotypes

**Figure 3:**
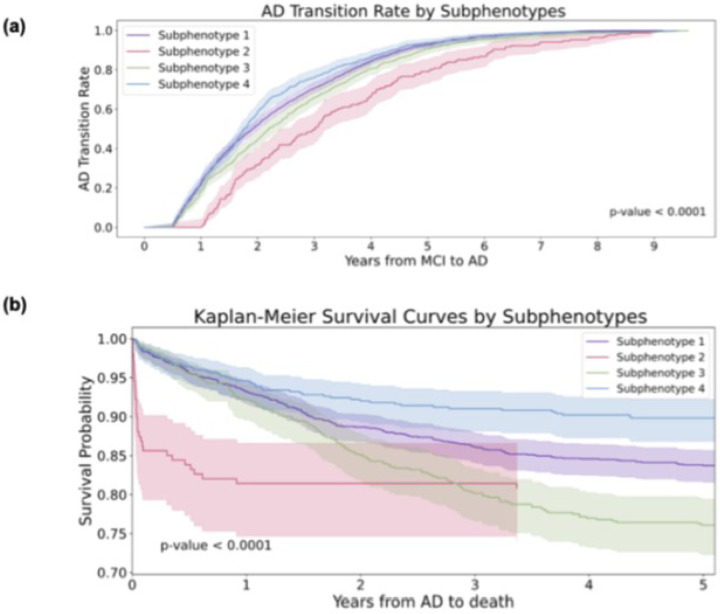
Visualization of Mild Cognitive Impairment (MCI) to Alzheimer’ Disease (AD) progession subphenotype. (a) MCI to AD transition rates by subphenotyeps. (b) Kaplan-Meier survival curves by subphenotypes.

**Figure 4:**
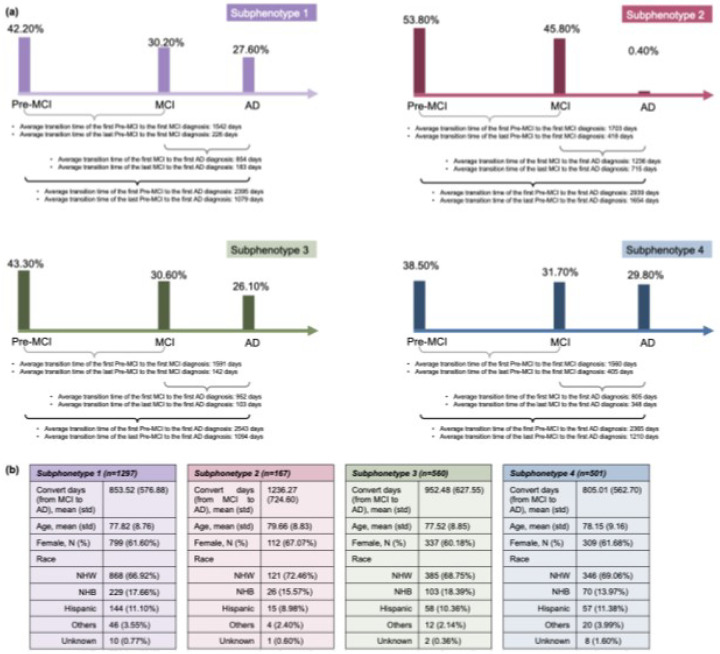
Characteristics of the AD progression subphenotypes. (a) Average transition times across the stages of AD in the four subphenotypes. (b) Demographics of each subphenotype.

Through the K-selection procedure by the combination of quantitative and qualitative analysis (**supplement section 1**), we determined that the optimal number of clusters was four (K = 4). Figure 3-a shows the transition rates and days from MCI to AD across subphenotypes. Figure 3-b visualizes the Kaplan-Meier curve, showing the 5-year survival rates for each subphenotype after AD diagnosis. There is a notable difference (p < 0.001) among the various subphenotypes. Figure 4-a shows detailed AD progression information among phenotypes. Patients in subphenotype 1 (S1) and S4 are characterized by faster progression, with average times from the initial record to the first AD diagnosis being 2, 395 days and 2,365 days, respectively. Conversely, individuals in S2 and S3 exhibit a more stable and slower progression to AD, with average transition times from the first EHR record to the first AD diagnosis being 2,939 days and 2,543 days, respectively. Figure 4-b illustrates the demographic statistics for each subphenotype; S1 includes a higher number of patients (n = 1,297) than the others, while the patients in these four subphenotypes share comparable demographic distributions. When considering the MCI to AD transition rate, S1 (mean: 854 days; std: 577 days) and S4 (mean: 805 days; std: 563 days) progress more rapidly than S2 (mean: 1,236 days; std: 725 days) and S3 (mean: 952 days; std: 628 days), however with large variations within each subphenotype.

**Figure 5:**
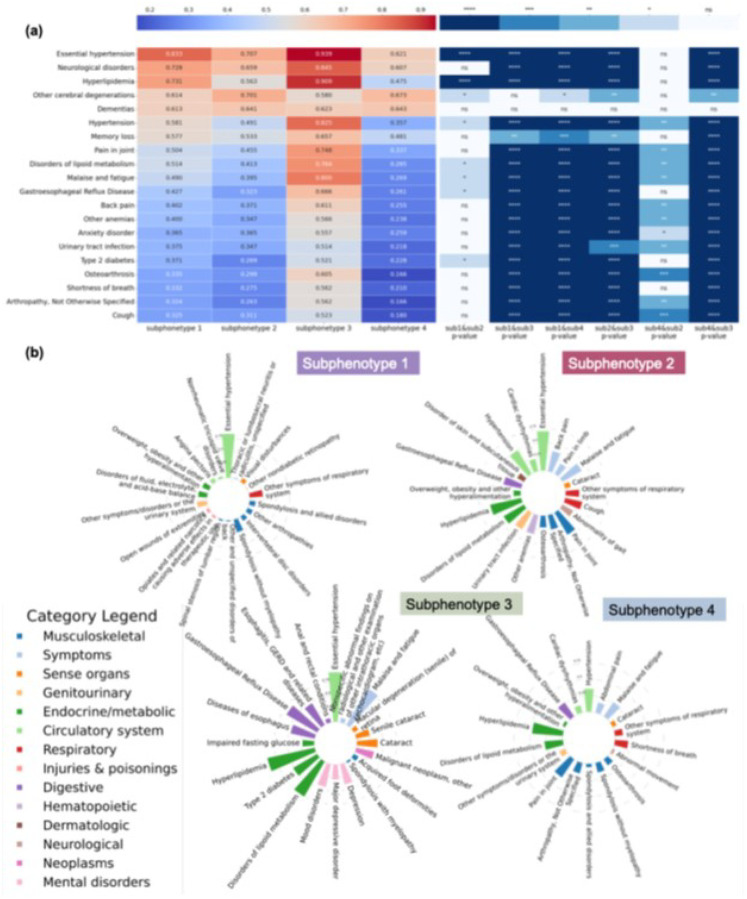
Clinical characteristics of the AD progression subphenotypes. (a) Heatmap of the top 20 features with the highest prevalence in the cohort across the four subphenotypes, along with p-value comparisons between each pair of subphenotypes. (b) Most correlated comorbidities and their prevalence for each subphenotype.

Figure 5 plots the unique clinical characteristics of each cluster. To gain insights into the features differentiating these subphenotypes, we first selected the top 20 features with the high percentage in the study cohort and then examined their prevalence within each subphenotype (left panel of Fig. 5-a). The analysis revealed that essential hypertension was the most prevalent disease among all the subphenotypes. Additionally, S4 included patients with lower prevalence, while S3 comprised patients with higher prevalence. We performed statistical analysis using Chi-square tests to examine the significant differences between pairs of subphenotypes. The corresponding p-values are presented in the right panel of Fig. 5-a. Notably, significant differences were observed between paired subphenotypes, with S1 being relatively closer to S2. Additionally, in Fig. 5-b, we highlight the top (filtered by p-value < 0.05) for each subphenotype, and the bar indicates the prevalence of specific factors within each subphenotype. Diseases of the circulatory system were common among all subphenotypes; overlapping features were observed, particularly conditions related to the musculoskeletal and endocrine/metabolic systems. S1 and S4, the faster progression subphenotypes, were linked to common comorbidities associated with AD, including cardiovascular, gastrointestinal, and musculoskeletal diseases. S2 and S3 showed relatively stable AD progression but had lower survival rates within five years after an AD diagnosis, with a higher prevalence and variety of associated comorbidities.

### Predictability and interpretability of the identified subphenotypes

**Figure 6:**
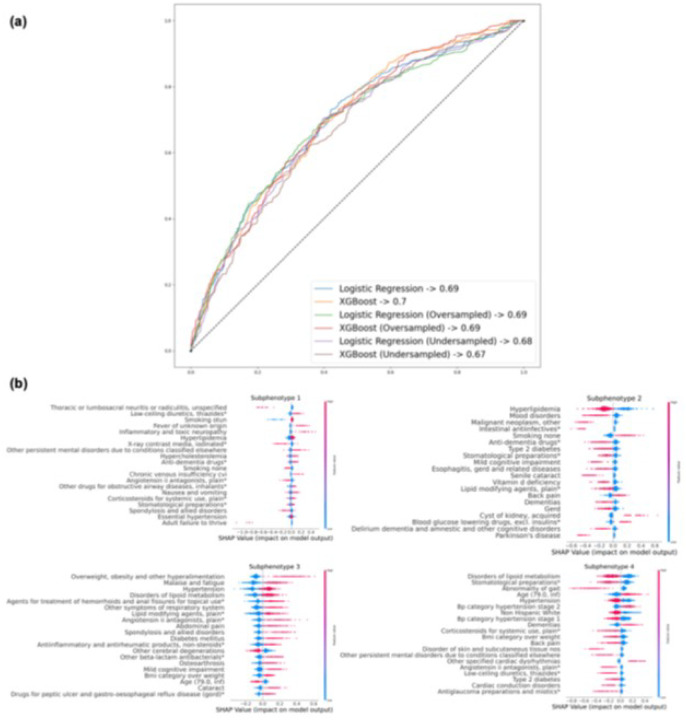
Comparative assessment of the predictability and interpretability of XGBoost and linear models in predicting AD subphenotypes. (a) Model performance for predicting progression subphenotypes based on encounter information. (b) Analysis of feature importance in XGBoost using SHAP values.

Figure 6-a illustrates the performance of the prediction models built to classify one of the four subphenotypes based on the information before the MCI diagnosis date, using different prediction algorithms (e.g., linear models vs. XGBoost) and resampling strategies (e.g., oversampling vs. undersampling) to address data imbalance. Our results showed that all models delivered fair performances, with Area Under the Receiver Operating Characteristic (AUROC) ranging from 0.67 to 0.7, where XGBoost showed comparable predictive performance with logistic regression. Regarding resampling methods, non-resampling, oversampling, and undersampling all yielded similar results. Figure 6-b presents the SHAP values of the XGBoost model, which predicts the likelihood of patients being classified into different subphenotypes. Across the four subphenotypes, the key factors influencing classification include the patient’s age, existing neurological conditions (e.g., memory loss), and current dementia stage (e.g., Pre-MCI, MCI, or dementia due to AD). Specifically focusing on S4, considered the most rapid progression subphenotype, the model underlined the fact that being older than 79, experiencing hypertension stage 2,^53,54^ and being on dementia medications are key predictors of being in this subphenotype, consistent with findings in the existing literature.^55^

## Discussion

We developed a novel outcome-oriented GNN framework that naturally (1) models sequences of events in longitudinal EHRs as directed graphs, and (2) captures similar patient encounters through directed edges. This framework enables GNNs to generate representations across patients with similar characteristics while considering the changes in individual patients’ health conditions. Utilizing time-series K-means clustering on the representations learned from GNNs, our approach can effectively model nuanced similarities in disease progression patterns. We used large collections of EHRs from the OneFlorida + network and identified 2,525 patients with MCI over an observation period of up to 10 years (**eTable 1**). Our results demonstrated that our proposed framework holds promise in detecting predictable AD progression subphenotypes, providing valuable and explainable insights into the development of the disease.

Several studies (Table 1) have focused on disease progression subtyping. However, most of this work either does not incorporate relevant clinical outcomes (e.g., the disease continuum) or is limited in modeling the similarities in progression trends and patterns among patients. As a result, these approaches struggle to effectively capture disease progression characteristics, reducing their utility for both clinicians and patients. For example, Xu et al. proposed an LSTM-based framework to define progression states and identified two distinct progression patterns within MCI cohorts, without considering progression pathways in similar patients.^23^ Song et al. introduced the DisEase PrOgression Trajectory (DEPOT) approach to model cancer-related chronic kidney disease (CKD) progression trajectories from electronic medical records.^56^ Nagamine et al. employed a cluster-based approach to understand the real-world manifestation and progression of heart failure by constructing disease states from clinical notes.^57^ Additionally, Chowdhury et al. encoded longitudinal patient EHRs into a graph structure and applied a graph transformer for drug response prediction. The learned embeddings from the graph transformer were then used to stratify patients into subgroups.^58^ In contrast to these existing methods, our framework leverages a DPG to model patient longitudinal EHRs as a directed graph, linking visits with similar clinical characteristics, which serves as a foundation for GNNs to learn comprehensive patient progression patterns. Furthermore, our framework adopts an outcome-oriented strategy to guide the representation learning process, ensuring that the extracted features are both clinically meaningful and predictive of the progression from MCI to AD. By integrating these key components, our method enhances the interpretability of disease progression, offering deeper insights into patient trajectories and supporting more informed clinical decision-making.

Using the GNN-based framework and time-series clustering, we identified four subphenotypes of patients with distinct progression patterns from MCI to AD. These patterns suggest that AD does not follow uniform transitions of disease states but rather exhibits heterogeneous progression pathways, aligning with the existing research^22,59–61^. For instance, Geifman et al.^59^ identified three clinical phenotypes of AD from clinical trials, each following a distinct trajectory: slow decline, severely impaired yet slow decline, or rapid decline. However, this study did not explore the clinical characteristics of these subtypes in detail, such as the correlation between comorbidities and each subtype. In contrast, Xu et al.^22^ identified four probable AD and related dementia subphenotypes characterized by associated conditions like cardiovascular diseases, mental health issues, diabetes, and sensory impairments. Nevertheless, this study did not elaborate on the progression patterns of these subphenotypes. In 2023, Xu et al.^23^ further defined two main distinct progressions within the MCI cohorts–one leading to AD and one that did not. The pathway leading to AD was notable for significant differences in symptoms such as memory loss, various dementias, and articular cartilage disorders, which are typical in AD cases. Garg et al.^62^ characterized the progression from MCI to AD into four categories with different health conditions using AD cohort study data from Mayo Clinic Study of Aging (MCSA). Compared to these studies, our framework identified four progression subphenotypes from MCI to AD from routine care records, providing detailed clinical characteristics of each, including comorbidities across various systems like nervous, musculoskeletal, cardiovascular, alimentary tract and metabolic, genitourinary, and sensory systems. We also outlined the distinct rates of progression for each subphenotype. A better understanding of these distinct disease progression patterns could help the exploration of more personalized and potentially effective treatment strategies, potentially slowing or preventing their progression to AD. Our approach can not only help identify (1) patients at a higher risk of AD progression, years before they reach the clinical stage, where early-stage treatments^63,64^, such as anti-amyloid drugs aducanumab and lecanemab (approved by the FDA for targeting AD’s underlying biology) might be more effective, but also (2) their potential disease progression pathways, so that providers and patients can better plan potential treatment strategies (e.g., the management of comorbidities and symptoms).

The subphenotypes we identified, showcasing variations in traits linked to disorders–primarily in musculoskeletal, circulatory, endocrine/metabolic, digestive, and sensor systems, as well as in their progression rates, provide important insights. Hypertension,^53,54^ often considered a risk factor for cognitive decline and AD, tied to all progressing subphenotypes. Disorders related to lipoprotein metabolism^65^ and symptoms such as malaise and fatigue were associated with three subphenotypes. Studies^66,67^ suggested that targeting APOE^68,69^, a key factor in lipid metabolism, could lead to developing treatments that may help AD patients. Managing symptoms, for example, through the therapeutic use of nicotinamide adenine dinucleotide (NAD)^70^, holds the potential to benefit patients with AD as well as those experiencing chronic fatigue syndrome. The rapidly progressing subphenotypes S1 and S4 were mainly associated with musculoskeletal and cardiovascular conditions, including cardiac arrhythmias.^71–73^ Managing cardiac arrhythmias could potentially slow cognitive decline in AD by reducing the risk of strokes and other cardiovascular complications.^73^ Furthermore, due to a lower prevalence of comorbidities compared to S2 and S3, these subphenotypes also exhibited lower five-year survival rates. For the stable progression subphenotype (S2), in addition to common comorbidities, we found associations with conditions such as urinary tract infections (UTIs),^74,75^ hematopoietic disorders, and neurological diseases. UTIs, in particular, were linked to cognitive impairments in individuals with MCI or AD, often manifesting as delirium and exacerbating dementia symptoms. However, treating UTIs may lead to some cognitive improvements. Our findings indicate that S3, a subgroup with a high prevalence of mental health conditions (e.g., mood disorders and depression), digestive disorders (e.g., esophageal diseases^76^), and neoplasms, exhibited a slower AD progression rate. However, while S2 and S3 demonstrated more stable AD progression patterns, their high prevalence of multiple comorbidities contributed to lower five-year survival rates compared to other subphenotypes. Understanding these diverse subphenotypes provides valuable insights into different patient subgroups with distinct characteristics. This knowledge can be leveraged for post-trial analysis, optimizing patient recruitment, and assessing drug effectiveness.

### Strengths and Limitations

Our study has several significant clinical implications. First, the proposed framework enables a more precise AD diagnosis by identifying unique progression pathways, each characterized by distinct clinical factors such as comorbidities and medication use. Second, recognizing various progression subphenotypes is very valuable for understanding the disease’s progression in AD patients, offering potential prognoses of AD, which is vital for informing future care and support planning. Finally, since each subphenotype may respond differently to treatments, discerning and testing these differences allows more targeted and effective therapeutic options if proven true in future trials, thus aiding in developing personalized treatment strategies.

Our study has two limitations. First, the analysis conducted in our study was based on a cohort of MCI and AD patients mainly in Florida, Georgia, and Alabama; this limited geographical scope might affect the generalizability of our findings. However, our real-world AD patients from OneFlorida + were highly diverse, including both rural and urban populations, and reflected the demographic changes (a high prevalence of racial-ethnic) minorities, occurring across the US. Nevertheless, future research should aim to enhance our proposed framework’s generalizability utilizing data from different geographic regions. Second, while the AUROC indicates an acceptable level of performance, other metrics suggest only fair predictive capability. Predicting progression subphenotypes remains a challenging task, and traditional models such as linear models and XGBoost struggle to achieve high accuracy. Additionally, the current subphenotyping prediction model is data-driven. Considering a knowledge-driven modeling approach may improve subphenotype prediction methods.

## Conclusion

We proposed a novel outcome-oriented GNN framework for identifying AD progression subphenotypes using EHRs. The four subphenotypes suggest that AD exhibits heterogeneous progression pathways rather than follow uniform transitions of disease states. These subphenotypes providing valuable and explainable insights for the development of the AD.

## Figures and Tables

**Table 1:** Relevant study comparison

	Disease Focus	Key method		Outcomes
		Representation Learning	Clustering	
Xu et al.^23^	Alzheimer’s disease (AD) progerssion	Long short-term memory-based deep learning	Hierarchical Agglomerative Clustering	Identify AD progression states and summarize progession patterns
Song et al.^56^	Chronic kidney disease (CKD) progression	GraphSAGE	DDRTree	Maps diverse CKD progression paths and cancer risks
Nagamine et al.^57^	Heart Failure	NLP feature extraction	K-Means	Identify heart failure disease states
Chowdhury et al.^58^	Heart Failure drug response	Graph Neural Network + Transformer	K-Means	Identifies HF subtypes with differential drug responses
Ours	Mild cognitive impairment (MCI) to AD progerssion	Outcome-oriented Graph neural networks	Time series KMeans	Identify MCI to AD progression subphenotypes

## Data Availability

The data presented in this study are available on request from the corresponding author. The data are not publicly available due to privacy restrictions.
